# UK Antimicrobial Registry: Virtual Registry-an innovative surveillance approach for monitoring the real-world use and effectiveness of newly licensed antimicrobials in Scotland

**DOI:** 10.1093/jacamr/dlag053

**Published:** 2026-04-15

**Authors:** Cosmika Goswami, Ebru Turgal, Tanja Mueller, Marion Bennie, Rebecca Parr, Gareth T Jones, R Andrew Seaton, David Jenkins, Ioannis Baltas, Jacqueline Sneddon, Jonathan A T Sandoe, Frances Garraghan, Nicholas M Brown, Gary J Macfarlane, Amanj Kurdi

**Affiliations:** Strathclyde Institute of Pharmacy and Biomedical Sciences, University of Strathclyde, Glasgow G4 0RE, UK; Strathclyde Institute of Pharmacy and Biomedical Sciences, University of Strathclyde, Glasgow G4 0RE, UK; Strathclyde Institute of Pharmacy and Biomedical Sciences, University of Strathclyde, Glasgow G4 0RE, UK; Strathclyde Institute of Pharmacy and Biomedical Sciences, University of Strathclyde, Glasgow G4 0RE, UK; Epidemiology Group, School of Medicine, Medical Sciences and Nutrition, University of Aberdeen, Aberdeen, UK; Epidemiology Group, School of Medicine, Medical Sciences and Nutrition, University of Aberdeen, Aberdeen, UK; British Society for Antimicrobial Chemotherapy, Birmingham, UK; Infectious Diseases Unit, Queen Elizabeth University Hospital, NHS Greater Glasgow and Clyde, Glasgow, UK; Clinical Microbiology, University Hospitals of Leicester NHS Trust, Leicester, UK; British Society for Antimicrobial Chemotherapy, Birmingham, UK; Department of Infection, Immunity and Inflammation, Institute of Child Health, University College London, London, UK; Department of Microbiology, University College Hospital, University College London Hospitals NHS Foundation Trust, London, UK; British Society for Antimicrobial Chemotherapy, Birmingham, UK; Healthcare Associated Infection Group, Leeds Institute of Medical Research, University of Leeds and Leeds Teaching Hospitals NHS Trust, Leeds, UK; British Society for Antimicrobial Chemotherapy, Birmingham, UK; Clinical Microbiology & Public Health Laboratory, Cambridge University Hospitals NHS Foundation Trust, Cambridge, UK; Epidemiology Group, School of Medicine, Medical Sciences and Nutrition, University of Aberdeen, Aberdeen, UK; Strathclyde Institute of Pharmacy and Biomedical Sciences, University of Strathclyde, Glasgow G4 0RE, UK; College of Pharmacy, Al-Kitab University, Kirkuk 36015, Iraq; Department of Public Health Pharmacy and Management, School of Pharmacy, Sefako Makgatho Health Sciences University, Pretoria, South Africa; College of Pharmacy, Hawler Medical University, Erbil, Kurdistan Region, Iraq

## Abstract

**Background:**

Monitoring the real-world use of recently licensed antimicrobials (RLAs) is critical for antimicrobial stewardship. Traditional surveillance systems are resource-intensive and limited in scope.

**Objectives:**

The UK Antimicrobial Registry: Virtual Registry (UKAR:V) was established to determine whether routinely collected electronic healthcare data can generate robust, national-level evidence on the utilization, effectiveness and safety of RLAs in Scotland.

**Methods:**

This registry used linked data from Scotland’s Hospital Electronic Prescribing and Medicines Administration system and national datasets. Adults (≥18 years) prescribed any of 11 RLAs (cefiderocol, ceftazidime/avibactam, ceftolozane/tazobactam, meropenem/vaborbactam, imipenem/cilastatin/relebactam, eravacycline, ceftaroline, ceftobiprole, dalbavancin, delafloxacin, oritavancin) between June 2019 and June 2023 were included. Descriptive analyses summarized patient characteristics, prescribing patterns, infection types, microbiology results and outcomes.

**Results:**

Overall, 308 patients received 353 RLA prescriptions. Dalbavancin was commonly prescribed (70.5%), followed by ceftazidime/avibactam (13.3%). Microbiology results were available for 35% of patients. *Pseudomonas aeruginosa* (43.7%) and *Klebsiella pneumoniae* (19.5%) were the most common isolates for Gram-negative RLAs, while *Staphylococcus aureus* (50%) predominated among Gram-positive RLAs. Gram-negative RLAs were mainly used for severe respiratory and sepsis cases, whereas dalbavancin was used for skin, soft-tissue and device-related infections. Median treatment duration ranged from 7 to 12 days for Gram-negative RLAs and one dose for dalbavancin. Twenty-eight-day readmission was 25%–40% for Gram-negative RLAs and 29.8% for Gram-positive RLAs, while 6-month relapse ranged from ∼38% to 67% and 51.7%, respectively. No major linkage issues/failures were identified.

**Conclusions:**

UKAR:V shows that linked electronic data can support real-world RLA surveillance. With appropriate data linkage, this model offers a scalable, low-burden approach to monitoring utilization/outcomes providing a sustainable foundation for stewardship/policy and assessment of innovative reimbursement models.

## Introduction

Antimicrobial resistance (AMR) continues to pose one of the most pressing threats to global public health, undermining decades of progress in infectious disease control and modern medical care.^1,2^ In 2019, bacterial AMR was directly responsible for an estimated 1.14 million deaths worldwide and associated with nearly 5 million deaths overall.^3^ Without urgent action, global mortality attributable to AMR is projected to rise by almost 70% by 2050.^3^ This growing burden is driven by the increasing prevalence of multidrug-resistant Gram-negative pathogens such as *Escherichia coli*, and *Klebsiella pneumoniae*, which severely constrain therapeutic options and necessitate the use of last-line recently licensed antimicrobial agents (RLAs).^1,4^ These RLAs include new beta lactam and beta lactamase inhibitor combinations such as ceftazidime/avibactam, ceftolozane tazobactam, meropenem vaborbactam and imipenem relebactam, which were designed to improve stability against key carbapenemases and resistant phenotypes.^4,5^ Other innovations include the siderophore cephalosporin cefiderocol, which achieves enhanced activity against difficult-to-treat non-fermenters by utilizing bacterial iron transport pathways.^6^ For Gram-positive infections, long-acting lipoglycopeptides such as dalbavancin and oritavancin, as well as newer agents, like delafloxacin, ceftaroline and ceftobiprole, offer extended half-lives, broad activity against resistant staphylococci and the possibility of outpatient therapy.^7,8^ While clinical trials underpin the licensing of such agents, their restrictive inclusion criteria often exclude patients with complex comorbidities, or prior antimicrobial exposure.^4,5^ Consequently, post-marketing data on the real-world effectiveness, safety and utilization of RLAs continues to be scarce. Robust surveillance systems capable of capturing their use across diverse healthcare settings are essential to optimize prescribing, support antimicrobial stewardship and inform both clinical guidance and national funding models such as the UK’s value-based ‘subscription’ scheme for new antimicrobials.^4,9^

To address these persistent gaps in real-world evidence for newly licensed antimicrobials, the UK Antimicrobial Registry (UKAR) was established in 2021.^4^ It consists of a UKAR: Hospital Registry (UKAR:H) that captures detailed clinical and microbiological data on agents initially included in the NICE subscription model pilot (cefiderocol and ceftazidime/avibactam) and now also ceftolozane/tazobactam, meropenem/vaborbactam, imipenem/cilastatin/relebactam, eravacycline, ceftaroline, ceftobiprole, dalbavancin, delafloxacin and oritavancin. UKAR supports national stewardship, but the UKAR:H model depends on manual data entry and prospective recruitment, limiting scalability.^4^ The UKAR: Virtual Registry (UKAR:V) was developed in Scotland to exploit NHS Scotland’s linked data capabilities and to complement UKAR:H by using routinely collected electronic health records to enable automated, large-scale surveillance of antimicrobial utilization, effectiveness and safety.^4^ It is important to note that in this study, the term ‘Recently Licensed Antimicrobials (RLAs)’ is used to describe a group of newer antimicrobial agents included within the UKAR programme and the NICE subscription model, which focus on antimicrobials developed to treat multidrug-resistant pathogens. Although some of these agents, such as ceftazidime–avibactam, were licensed earlier, they remain part of the group of newer targeted antimicrobials for which ongoing real-world surveillance of utilization and outcomes is required.

Scotland’s integrated digital infrastructure, which links prescribing, microbiology and outcomes data through a single patient identifier, provides an efficient setting to evaluate a virtual registry model. Linked national datasets such as the Scottish Morbidity Records, the Electronic Communication of Surveillance Scotland microbiology system and national prescribing datasets have previously been used to support pharmacoepidemiological research, health service evaluation and antimicrobial stewardship analyses at population scale.^10–13^ These linked data resources allow the study of medicines use and outcomes in routine clinical practice, including patients with complex comorbidities or prior treatment exposures who are often underrepresented in Phase III clinical trials. This capability is particularly important for RLAs, where post-marketing evidence is needed to understand their effectiveness and safety in real-world populations. In addition, antimicrobial prescribing in Scotland is supported by a nationally coordinated stewardship programme led by the Scottish Antimicrobial Prescribing Group (SAPG), which promotes adherence to evidence-based treatment guidance, monitors antimicrobial use across NHS Scotland and supports surveillance of AMR and prescribing practices.^14^ This coordinated stewardship framework helps ensure that antimicrobial prescribing generally aligns with national guidance, thereby providing a structured context in which real-world utilization and outcomes of newly licensed antimicrobials can be evaluated. Such integrated stewardship systems and national data infrastructures are not universally available, particularly in some low- and middle-income settings where antimicrobial prescribing practices and surveillance capacity may be more variable.^15^  ^,16^ The Scottish context therefore offers an informative model for how linked health data and national stewardship programmes can jointly support evidence generation and responsible antimicrobial use. While several countries, including Denmark, have similarly advanced infrastructures,^17,18^ Scotland offers a robust environment to test whether routine electronic data can reproduce the depth of a UKAR:H. The primary aim of this study was to assess the feasibility of constructing a national UKAR:V using routinely collected electronic healthcare data and to evaluate its capacity to generate reliable surveillance outputs for RLAs. While the system was designed to complement the UKAR:H, formal comparison with the UKAR:H was beyond the scope of this initial phase and will be undertaken in future phases once comparable data become available. Through descriptive analyses of utilization patterns, microbiology results and clinical outcomes, the study also aimed to demonstrate the analytical capability of the UKAR:V platform to support future clinical effectiveness research once larger datasets become available.

## Materials and methods

### Study design, data source, and study population

This study used Scotland’s national data infrastructure, which enables deterministic linkage of individual health records through a unique NHS identifier. Inpatient prescribing and administration data were obtained from the Hospital Electronic Prescribing and Medicines Administration (HEPMA) system^19^ implemented across six of Scotland’s 14 health boards during the study period and covering around 65% of the population. HEPMA records medicine name, dose, route, frequency and administration time, allowing accurate measurement of antimicrobial exposure. To capture broader clinical context, HEPMA data were linked to multiple routinely collected datasets, including the Scottish Morbidity Records (SMR01 for general acute inpatient and day cases, SMR02 for maternity and SMR06 for cancer registrations)^20^; the Electronic Communication of Surveillance Scotland (ECOSS)^21^ dataset containing microbiology results such as organism identification, specimen type, and antimicrobial susceptibility; the National Records of Scotland (NRS) mortality data; and specialty datasets including the Scottish Intensive Care Society Audit Group (SICSAG)^22^ and Scottish Renal Registry (SRR).^23^ The routinely collected datasets used in this study, including HEPMA prescribing records, Scottish Morbidity Records (SMR), ECOSS microbiology data and national mortality records, are well-established national data resources that have been widely used in pharmacoepidemiological and health services research in Scotland. Previous studies using these linked datasets have demonstrated high levels of data completeness, accuracy and internal consistency when examining antimicrobial prescribing, treatment duration and infection-related outcomes in routine clinical practice.^10–13^ Deterministic linkage through the unique patient identifier minimizes linkage errors and allows consistent tracking of patient records across multiple healthcare datasets. Building on this established infrastructure, additional data cleaning and validation procedures were undertaken in the present study to ensure consistency across datasets. These included restricting the analysis to administered prescriptions, consolidating duplicate prescribing entries generated when dose or formulation changes occurred within the same treatment episode^11^ and verifying consistency between prescribing episodes and hospital admission records. Records with incomplete or inconsistent admission or discharge information were excluded to maintain analytical integrity, while variables with partial data availability (such as microbiology testing) were retained with denominators clearly reported to reflect data completeness. The study received approval from the NHS Research Ethics Committee and the Public Benefit and Privacy Panel for Health and Social Care (Reference 2223-0155).

The study population included all adults (≥18 years) prescribed at least one RLA during a hospital admission within HEPMA-enabled Scottish health boards between January 2019 and June 2023. Eligible agents comprised 11 RLAs active against multidrug-resistant Gram-positive and Gram-negative pathogens: cefiderocol, ceftazidime/avibactam, ceftolozane/tazobactam, meropenem/vaborbactam, imipenem/cilastatin/relebactam, eravacycline, ceftaroline, ceftobiprole, dalbavancin, delafloxacin and oritavancin.^4^ Patients were identified through HEPMA prescribing records, with only administered prescriptions retained to ensure accurate measurement of drug exposure. Exclusions were applied to individuals aged under 18 years, those ordinarily resident outside Scotland, who lack the unique identifier required for deterministic linkage and records with incomplete admission or discharge data. The resulting cohort represents a population-based sample of hospitalized adults treated with RLAs across health boards covering approximately two-thirds of the national population during the study period. Each RLA treatment episode was treated as the index event and linked across datasets to capture demographic, clinical and outcome information, with follow-up extending to 12 months to ascertain readmission, relapse and mortality.

### Study outcomes

The primary outcomes of interest were the utilization patterns of RLAs and associated prescribing behaviours in real-world clinical practice. Data were also collected on patient demographics [age, sex, ethnicity and socioeconomic status using the Scottish Index of Multiple Deprivation (SIMD)^24^], comorbidity profiles [quantified Charlson Comorbidity Index (CCI)^25^], infection types and treatment regimens. Each regimen captured the RLA prescribed, its type, dose, route and frequency, with specific attention to concomitant antibiotics, defined as non-RLA antimicrobial agents administered concurrently with the RLA (i.e. overlapping administration dates). Microbiology testing was also recorded, defined as any diagnostic culture or sensitivity test conducted within 7 days before or after initiation of RLA treatment.

Secondary outcomes assessed clinical effectiveness and safety, including hospital readmission within 28 and 56 days, relapse of the index infection at 6 and 12 months and all-cause mortality during treatment, at 28 days and up to 12 months post-treatment. These outcomes were used as indicators of patient trajectory following RLA treatment rather than as diagnosis-specific measures of clinical effectiveness. Treatment-related measures [RLA duration, length of hospital stay and initiation within intensive care unit (ICU) settings] were evaluated as indicators of treatment intensity and clinical complexity. Because HEPMA is not used within ICU prescribing systems, ICU initiation was identified through linkage with SICSAG, where RLA administration overlapped with an ICU admission, or through SMR01 admissions associated with an ICU specialty code. Consistent with the feasibility aims of this pilot study, we also examined feasibility outcomes, including the proportion of eligible prescriptions captured within HEPMA; completeness of key demographic, prescribing, microbiology and outcome variables; the extent of missing data requiring exclusion; and the availability of microbiology and susceptibility information to support linkage-based surveillance.

### Data analysis

Descriptive statistics were used to summarize patient demographics, comorbidity profiles, infection types and prescribing patterns. Categorical variables were reported as frequencies and percentages and continuous variables as means (standard deviation) or medians (interquartile range) according to distribution. Results were stratified by Gram-positive and Gram-negative RLAs where relevant to illustrate class-specific prescribing trends. Microbiology data were examined to identify organisms associated with RLA use. Concomitant antibiotic use, treatment duration and length of hospital stay were evaluated as indicators of treatment complexity and clinical burden. Analyses of readmission, relapse and mortality were descriptive and non-comparative due to small sample sizes, with time-to-event outcomes to assess feasibility rather than infer causality. Formal hypothesis testing was not conducted, as the aim was to demonstrate the analytical capability of the UKAR:V and establish a framework for future inferential analyses.

## Results

**Figure 1.**
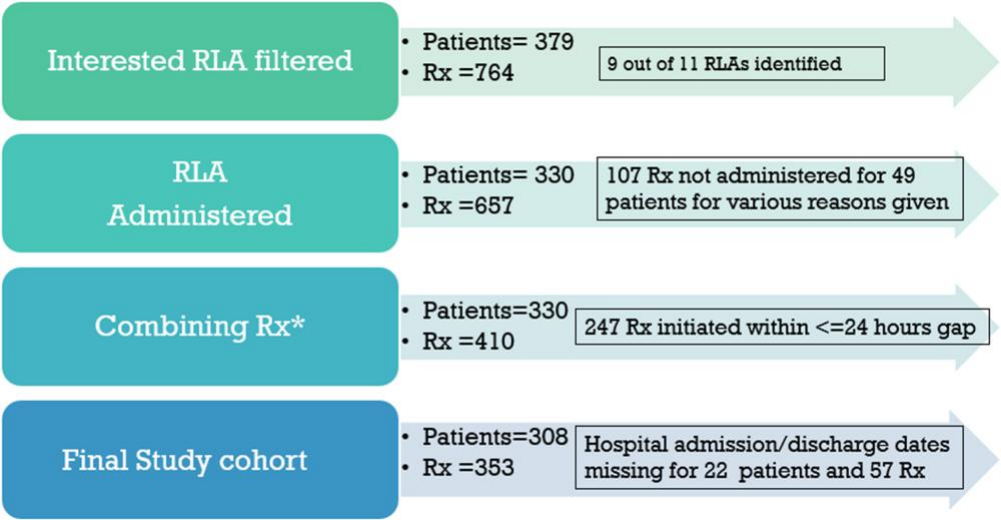
Four-step data cleaning process of RLA used to define the study population. Step 1 involved identifying all RLA prescriptions issued between June 2019 and June 2023. In Step 2, prescriptions that were not administered were excluded; *In Step 3, prescriptions for the same RLA initiated within 24 h were combined because the system records a new entry whenever dose, frequency, route or formulation changes occur within the same hospital episode; Step 4 retained only those prescriptions with complete hospital admission and discharge date information.

Between June 2019 and June 2023, 379 patients across Scotland received 764 RLA prescriptions. After excluding 107 prescriptions that were not administered and consolidating multiple entries for the same agent initiated within 24 h (necessary because HEPMA records a new entry whenever dose, frequency, route or formulation changes),^11,19^ the dataset included 330 patients and 410 prescriptions (Figure 1). Following removal of records with incomplete admission or discharge data, the final cohort comprised 308 patients with 353 administered RLA prescriptions (Figure 1).

The mean age at first prescription was 54 years (SD 17.7), with 13% (40/308) aged over 70 years, and 55.2% (170/308) were male (Table 1). Most patients were of White ethnicity (77.6%, 239/308), and 40% (123/308) lived in the most socioeconomically deprived SIMD quintile. Comorbidity burden was generally low, with 65% (200/308) having a CCI of 0 and only 2% (5/308) a CCI ≥ 3 (Table 1). However, Gram-negative RLA recipients had higher comorbidity levels: 40.4% (40/99) had CCI ≥ 1, compared with 33.1% (84/254) among Gram-positive RLA recipients. Conversely, a greater proportion of Gram-positive RLA patients had no comorbidities (66.9%, 170/254) than those receiving Gram-negative agents (59.6%, 59/99), reflecting a higher comorbidity burden among the latter group.

**Table 1.** Baseline demographic and clinical characteristics of 308 patients prescribed 353 RLAs

Description	Total patients = 308		Total Rx = 353	
Age	Mean ± SD	54 ± 17.7	Gram-negative RLA (n = 99, 28%)	Gram-positive RLA (n = 254, 72%)
Description	Groups	N (%)	N (%)	N (%)
Age group (years)	18–40	118 (38.3%)	36 (36.4%)	61 (24%)
	41–50	55 (17.9%)	6 (6.1%)	53 (20.9%)
	51–60	44 (14.3%)	28 (28.3%)	40 (15.7%)
	61–70	51 (16.6%)	13 (13.1%)	37 (14.6%)
	70+	40 (13%)	16 (16.2%)	63 (24.8%)
Gender	Female	138 (44.8%)	47 (47.5%)	136 (53.5%)
	Male	170 (55.2%)	52 (52.5%)	118 (46.5%)
Ethnicity	Asian	5 (1.6%)	2 (2%)	1 (0.4%)
	White	239 (77.6%)	79 (79.%8)	198 (78%)
	Others	64 (20.8%)	18 (18.2%)	55 (21.6%)
SIMD (quintile)	1 (most deprived)	123 (40%)	47 (47.4%)	102 (40.2%)
	2	78 (25.3%)	26 (26.3%)	61 (24%)
	3	40 (13%)	10 (10.1%)	34 (13.4%)
	4	37 (12%)	11 (11.1%)	30 (11.8%)
	5 (least deprived)	30 (9.7%)	5 (5.1%)	27 (10.6%)
Charlson Comorbidity Index	0	200 (65%)	59 (59.6%)	170 (66.9%)
	1	84 (27%)	30 (30.3%)	64 (25.2%)
	2	19 (6%)	10 (10.1%)	17 (6.7%)
	≥3	5 (2%)	0 (0%)	3 (1.2%)

SD, standard deviation; SIMD, Scottish Index of Multiple Deprivation.

### Prescribing patterns

**Figure 2.**
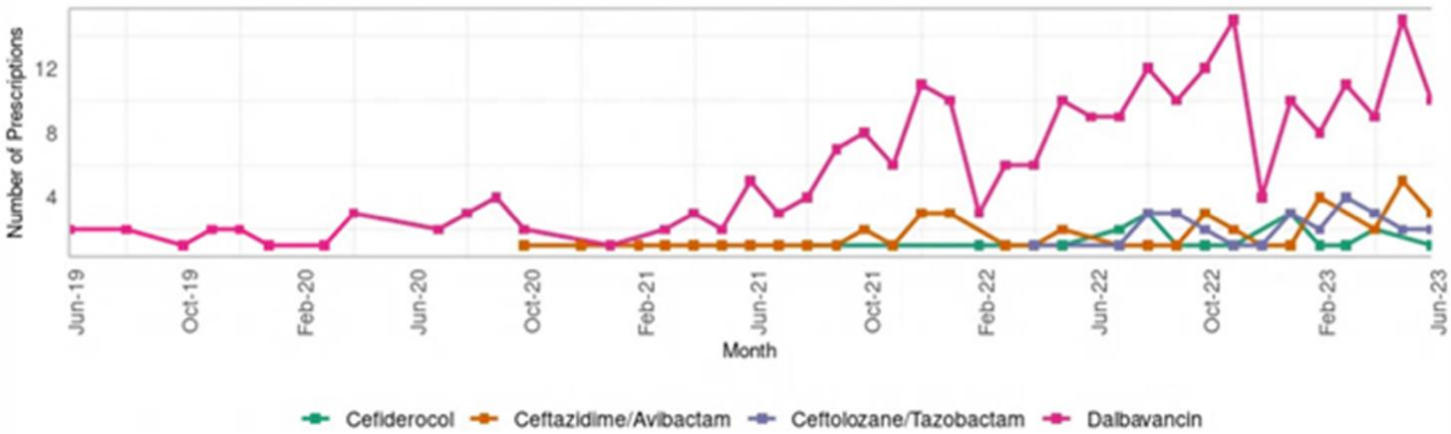
Monthly prescribing trends for selected RLAs in Scotland (June 2019–June 2023).

Of the 11 RLAs of interest, nine were identified in the HEPMA dataset; eravacycline and imipenem/cilastatin/relebactam were not prescribed during the study period. Four agents had sufficient prescribing frequency (≥5) for individual reporting (dalbavancin, ceftazidime/avibactam, ceftolozane/tazobactam and cefiderocol) and only these are described, in accordance with Public Health Scotland’s small-number suppression policy (Table 2). Dalbavancin was the most frequently used agent, accounting for 70.5% (249/353) of prescriptions, followed by ceftazidime/avibactam (13.3%, 47/353), ceftolozane/tazobactam (7.9%, 28/353) and cefiderocol (5.7%, 20/353). Prescribing patterns were broadly consistent across age and gender, though RLAs were more commonly prescribed for patients from more socioeconomically deprived groups, mirroring patterns seen at the patient level. To illustrate the capability of the UKAR:V to support longitudinal antimicrobial surveillance, temporal prescribing trends were examined for the four RLAs with sufficient prescribing frequency across the study period (Figure 2). Dalbavancin prescribing increased progressively from around 2021 onwards and remained the most frequently used agent throughout the study period. In contrast, prescribing of Gram-negative RLAs such as ceftazidime/avibactam, ceftolozane/tazobactam and cefiderocol remained comparatively lower but demonstrated intermittent increases over time.

**Table 2.** List of the 11 RLAs included in this study grouped by their spectrum of activity: Gram-positive agents and Gram-negative agents

Gram-negative agents	N (%)	Gram-positive agents	N (%)
Cefiderocol	20 (5.7%)	Ceftaroline	<5
Ceftazidime/avibactam	47 (13.3%)	Ceftobiprole	<5
Ceftolozane/tazobactam	28 (7.9%)	Dalbavancin	249 (70.5%)
Eravacycline	0 (0%)	Delafloxacin	<5
Imipenem/cilastatin/relebactam	0 (0%)	Oritavancin	<5
Meropenem/vaborbactam	<5		

Counts fewer than five are suppressed and reported as ‘<5’ in accordance with Public Health Scotland statistical disclosure control policies to minimize the risk of identifying individual patients.

### Infection types and microbiological findings

Microbiology results were available for 35% of patients (108/308), corresponding to 212 RLA prescriptions, indicating cases where a microbiology test (positive or negative) was recorded within the defined linkage window. The remaining 65% reflects instances where no microbiology record was linked within this window rather than confirmed negative results alone. Of these, 60% (128/212) related to Gram-negative RLAs and 40% (84/212) to Gram-positive agents (Table 3). Among Gram-negative infections, *Pseudomonas aeruginosa* (43.7%, 56/128) and *Klebsiella pneumoniae* (19.5%, 25/128) were the most frequent organisms. Gram-positive RLAs, predominantly dalbavancin, were mainly associated with *Staphylococcus aureus* (50%, 42/84) and *Enterococcus* spp. (12%, 10/84). Clinical indications varied by antimicrobial agents. Dalbavancin (249/353 prescriptions) was used for a wide range of infections, over 60 categories, with cellulitis (12%; 30/249), device-related infections (9.2%; 23/249) and osteomyelitis (7.6%; 19/249) most common. In contrast, Gram-negative RLAs (57/353) were typically used for respiratory infections, burn wounds, device- or surgery-associated infections and sepsis (Table 3).

**Table 3.** Distribution of bacterial isolates and infection types associated with selected Gram-positive and Gram-negative RLAs, presented as counts and percentages

		N (%)
Bacterial isolates for the 212 recently licensed antimicrobials (RLAs) prescriptions^a^		
Gram-negative	*Klebsiella pneumoniae*	17 (29%)
Cefiderocol (n = 59, 27.8%)	*Pseudomonas aeruginosa*	17 (29%)
	Others	25 (42%)
Ceftazidime/avibactam (n = 48, 22.6%)	*Pseudomonas aeruginosa*	24 (50%)
	*Klebsiella pneumoniae*	8 (17%)
	*Escherichia coli*	6 (13%)
	Others	10 (21%)
Ceftolozane/tazobactam (n = 21, 9.9%)	*Pseudomonas aeruginosa*	15 (71%)
	*Staphylococcus aureus*	5 (24%)
	Others	1 (5%)
Gram-positive		
Dalbavancin (n = 84, 39.6%)	*Staphylococcus aureus*	42 (50%)
	*Enterococcus* spp.	10 (12%)
	*Streptococcus pneumoniae*	8 (10%)
	Others	24 (29%)
Infection types for the 353 recently licensed antimicrobials (RLAs) prescriptions		
Gram-negative	Chronic pulmonary disease	5 (25%)
Cefiderocol (n = 20, 5.7%)	Burn	4 (20%)
	Pneumonia	2 (10%)
	Others	9 (45%)
Ceftazidime/avibactam (n = 47, 13.3%)	Cystic fibrosis	14 (29%)
	Sepsis	11 (23.4%)
	Acute pancreatitis	8 (17%)
	Others	14 (30.6%)
Ceftolozane/tazobactam (n = 28, 7.9%)	Cystic fibrosis	10 (36%)
	Bronchiectasis	7 (25%)
	Pneumonia	2 (7.1%)
	Others	9 (31.9%)
Gram-positive		
Dalbavancin (n = 249, 70.5%)	Cellulitis	30 (12%)
	Internal device/implants	23 (9.2%)
	Osteomyelitis	19 (7.6%)
	Others	177 (71.2%)
Prescriptions for the remaining five Gram-negative and Gram-positive RLAs (n = 9, 2.5%)	Others	9 (100%)

^a^Data on bacterial isolates are presented only for 212 prescriptions (n = 108 patients) where a microbiology test result was available.

### Treatment regimens and concomitant antibiotic use

Overall, 73.6% (260/353) of RLA prescriptions were issued alongside at least one additional antibiotic. Concomitant prescribing differed markedly between Gram-negative and Gram-positive RLAs. Among patients receiving Gram-negative RLAs (cefiderocol, ceftazidime/avibactam or ceftolozane/tazobactam), 98.9% (n = 92/93) were co-prescribed two or more additional antibiotics, with a median of three (IQR: 2–4). The most frequent co-prescribed agents included azithromycin, tobramycin, meropenem and metronidazole. By contrast, dalbavancin was generally prescribed with fewer concomitant antibiotics, with 66% (165/249) of dalbavancin used with a median of one concomitant antibiotic per course (IQR: 1-1), most often in a possible combination with flucloxacillin or vancomycin and occasionally clindamycin or rifampicin.

### Treatment duration and hospital stay

The median treatment duration varied notably between Gram-negative and Gram-positive RLAs (Table 4). Gram-negative agents, cefiderocol, ceftazidime/avibactam and ceftolozane/tazobactam, were typically administered for 7–12 days (IQR: 4–15), whereas dalbavancin was most often given as a single-dose course (equivalent to 7 days) (Table 4). The mean duration of concomitant antibiotic therapy was longer among Gram-negative RLAs (2–3 days) compared with dalbavancin (1 day). Median hospital stay also differed by spectrum of activity: patients receiving Gram-negative RLAs had longer admissions (9–18 days, IQR 3–42) than those treated with dalbavancin (8 days, IQR 4–19), consistent with the use of dalbavancin in facilitating early discharge or outpatient care (Table 4).

**Table 4.** Treatment duration and length of hospital stay associated with the 342 prescriptions for the four Gram-negative and Gram-positive RLAs included in the study

Recently licensed antimicrobials (n = 342)	Gram-negative (n = 93, 27.2%)			Gram-positive (n = 249, 72.8%)
	Cefiderocol (n = 20, 5.7%)	Ceftazidime/avibactam (n = 47, 13.4%)	Ceftolozane/tazobactam (n = 28, 8.2%)	Dalbavancin (n = 249, 72.8%)
Concomitant antibiotic duration; mean (±SD) days	3 (±1)	3 (±2)	2 (±1)	1 (±1)
treatment duration; median (IQR) days	7 (4–14)	8 (4–14)	12 (7–15)	1 (1–1)
Hospital length of stay; median (IQR) days	18 (8–42)	9 (3.5–23)	14 (9–26.5)	8 (4–19)

### Clinical characteristics and outcomes

Among the 301 patients receiving the four RLAs included in this study with enough data, approximately one-third were initiated in ICU settings, reflecting treatment complexity rather than ICU admission as a clinical outcome (Table 5). ICU prescribing was more common for Gram-negative RLAs (∼50.8%, n = 30/59), particularly ceftazidime/avibactam (71%.4, n = 20/28) and cefiderocol (56.3%, n = 9/16), consistent with their use in critically ill patients with multidrug-resistant infections. Outcome data demonstrated variation across antimicrobial classes. Readmission within 28 days occurred in 25%–40% of patients treated with Gram-negative RLAs and 29.8% (n = 72/242) among dalbavancin recipients, rising to ∼38% and 37.6% (91/242) respectively by 56 days. Relapse within 6 months was recorded in ∼38%–67% of Gram-negative RLA cases and 51.7% (125/242) among dalbavancin-treated patients, increasing to 58% at 12 months (Table 5). Twenty-eight-day all-cause mortality was 9.0% (n = 18/301) overall, with deaths primarily among patients receiving ceftazidime/avibactam and dalbavancin (nine each). No serious drug-related adverse events were identified; minor, self-limiting reactions such as mild headache, nausea and injection-site discomfort were occasionally reported.

**Table 5.** Summary of clinical outcomes for 301 patients treated with the four Gram-negative and Gram-positive RLAs included in the study

Recently licensed antimicrobials (n = 301)	Gram-negative (n = 59, 19.6%)			Gram-positive (n = 242, 80.4%)
	Cefiderocol (n = 16, 5.3%)	Ceftazidime/avibactam (n = 28, 9.3%)	Ceftolozane/tazobactam (n = 15, 4.9%)	Dalbavancin (n = 242, 80.4%)
ICU initiation^a^	9 (56.3%)	20 (71.4%)	<5 (<5%)	69 (28.5%)
Readmission within 28 days	4 (25%)	8 (28.6%)	6 (40%)	72 (29.8%)
Readmission within 56 days	5 (31.3%)	11 (39.3%)	9 (60%)	91 (37.6%)
Relapse within 6 months	6 (37.5%)	14 (50%)	10 (66.7%)	125 (51.7%)
Relapse within 12 months	6 (37.5%)	14 (50%)	11 (73.3%)	141 (58.3%)
28-day mortality	0 (0%)	9 (32.1%)	0 (0%)	9 (3.7%)

^a^Represents cases in which RLA was initiated in ICU settings.

## Discussion

This study shows that routinely collected electronic healthcare data can be used to create a virtual national registry for assess the real-world use of RLAs in Scotland. By linking HEPMA prescribing data with multiple national datasets, the UKAR:V offers a scalable, low-burden surveillance model that complements the manually curated UKAR:H.^4^ The analyses presented are intentionally descriptive because the primary objective of this first phase was to demonstrate the feasibility and surveillance capability of the virtual registry infrastructure rather than to conduct comparative effectiveness analyses. By integrating prescribing records with microbiology, hospitalization and mortality datasets, the platform enables systematic capture of antimicrobial utilization patterns, infection characteristics and patient outcomes across multiple health boards. The ability to reliably link and analyse these data elements illustrates the operational robustness of the underlying data infrastructure and establishes a foundation for future risk-adjusted comparative analyses, treatment pathway evaluations and pharmacoepidemiological clinical effectiveness studies once larger datasets become available. The reported outcomes demonstrate how UKAR:V can characterize real-world treatment trajectories and highlight its analytical potential for future large-scale evaluations of RLAs.

### Patterns of use and comparability with existing literature

Consistent with previous UKAR:H and international registry findings,^4,26,27^ dalbavancin was the most frequently used RLA, reflecting its long half-life, convenient once-weekly dosing and suitability for early discharge or outpatient management. Its widespread use across Scottish hospitals aligns with reports from other high-income settings where dalbavancin is increasingly adopted for complex skin, soft-tissue and device-related infections.^28^ The broad age and comorbidity range among dalbavancin recipients suggests pragmatic use in patients for whom prolonged inpatient therapy is undesirable. Use of Gram-negative RLAs (cefiderocol, ceftazidime/avibactam and ceftolozane/tazobactam) was less common and largely guided by microbiological confirmation of multidrug-resistant organisms, particularly *P. aeruginosa* and *K. pneumoniae*, consistent with international experience that these agents are reserved for targeted, high-risk scenarios.^26,27^ The frequent co-prescribing of additional antibiotics with Gram-negative RLAs likely reflects the clinical complexity of these cases and stewardship caution when managing severe or polymicrobial infections. In Scotland, dalbavancin use may also be influenced by geographical and service delivery factors. Several health boards cover large rural areas where long travel distances can limit access to hospital-based outpatient antimicrobial therapy services. In such settings, dalbavancin is often used to facilitate early discharge, reduce repeated hospital attendance and support care closer to home, factors that may contribute to its high utilization and warrant further investigation into regional prescribing patterns. The longitudinal prescribing trends identified in this study further demonstrate the surveillance capability of the UKAR:V. Temporal analyses showed increasing use of dalbavancin over the study period alongside more variable prescribing of Gram-negative RLAs. This pattern likely reflects a combination of increasing clinical familiarity with newer agents and the progressive implementation of HEPMA across Scottish health boards, which expanded the number of hospitals contributing prescribing data over time. The ability to capture such temporal changes highlights the value of linkage-based surveillance systems for monitoring uptake of newly licensed antimicrobials, identifying shifts in prescribing behaviour and evaluating the impact of stewardship interventions or policy changes.

### Comparison with UKAR:H findings

Although a direct comparison between UKAR:V and data collected in the UKAR:H was not one of the objectives of the current initial feasibility study, the prescribing patterns observed in our analysis are broadly consistent with findings reported from the first 20 months of recruitment to the UKAR:H, including over 600 patients across the whole of the UK.^29^ In that analysis, dalbavancin accounted for the majority of prescriptions among Gram-positive RLAs, while ceftazidime/avibactam, cefiderocol and ceftolozane/tazobactam were the most frequently used Gram-negative agents. These same agents also predominated in the UKAR:V. Furthermore, Gram-negative RLAs were most frequently prescribed for lower respiratory tract infections (44.8%), followed by systemic infections including sepsis (18.0%) and urinary tract infections (11.1%), while Gram-positive agents were primarily used for skin and soft-tissue infections (36.1%) and bone and joint infections (32.9%).^29^ These patterns are comparable to those observed in UKAR:V, where dalbavancin was commonly used for skin, device-related and bone infections, and Gram-negative RLAs were largely used for severe respiratory, device-related or systemic infections. Microbiological findings were also similar across the two datasets, with *P. aeruginosa* and *K. pneumoniae* predominating among Gram-negative infections and *S. aureus* among Gram-positive infections. Microbiology results were available for 35% of patients in the present analysis. As outlined in the limitations section, this partly reflects the defined microbiology capture window applied around RLA initiation to minimize misclassification of cultures unrelated to the index infection, which may have reduced the proportion of linked microbiology records within the dataset. Consequently, the observed proportion should be interpreted primarily as a reflection of data linkage constraints rather than as an indicator of incomplete microbiological investigation or inappropriate prescribing. In addition, some infections treated with RLAs, particularly those managed with dalbavancin for skin, soft-tissue or device-related infections, may be treated empirically in routine clinical practice without microbiological confirmation. Similar prescribing patterns have been reported in early analyses from UKAR:H, where a proportion of RLA prescriptions were initiated empirically or prior to definitive microbiological confirmation, reflecting the clinical need to initiate therapy promptly in patients with severe or complex infections.^29^ In addition, UKAR:H reported frequent use of concomitant or prior antibiotics in patients receiving Gram-negative RLAs (approximately two-thirds receiving combination therapy), reflecting the complexity of infections and stewardship-driven cautious deployment of these agents.^29^ A similar pattern of combination or sequential antibiotic therapy was observed in our study, supporting the interpretation that RLAs are typically introduced within broader treatment pathways rather than as isolated therapeutic interventions. These similarities provide preliminary reassurance that virtual registry approaches based on linked routine healthcare datasets may be capable of generating surveillance outputs that are broadly comparable to those produced by traditional prospective registries. At the same time, the UKAR:V model offers important additional advantages, including scalability, reduced reporting burden on clinicians and the ability to capture prescribing patterns across large populations using routinely collected data. Future work will formally compare the Scottish data from UKAR:H and the UKAR:V using overlapping study periods. Such analyses will help determine the extent to which virtual registry approaches can complement or augment traditional registries for national antimicrobial surveillance. It is also important to note that consistent with the primary objective of the study, our study did not assess the impact of newly licensed antimicrobial use on AMR patterns, nor did evaluate the appropriateness of prescribing or the clinical rationale underlying individual treatment decisions. Although detailed resistance mechanism information was not consistently available within the linked microbiology datasets, similar information has also not been comprehensively reported in early analyses of UKAR:H.^29^ Nevertheless, the organisms identified in association with Gram-negative RLAs, particularly *P. aeruginosa* and *K. pneumoniae*, are well recognized causes of multidrug-resistant infections for which these agents are typically reserved. These patterns are broadly consistent with findings from the first 20 months of the UKAR:H, where RLAs were predominantly prescribed for severe infections caused by resistant pathogens and frequently used following prior or concomitant antibiotic therapy.^29^

### Methodological advantages and feasibility of the virtual registry

The virtual registry model addresses several limitations of UKAR:H by removing the need for manual data entry, reducing reporting delays and capturing treatment events across all HEPMA-enabled hospitals, thereby improving coverage and representativeness. Deterministic linkage allows longitudinal follow-up of outcomes such as readmission, relapse and mortality without additional clinician workload, which is particularly valuable where trial evidence for newly licensed antimicrobials is limited or based on highly selected populations.^3,30^ Through integration of prescribing, laboratory and outcome data, UKAR:V demonstrates that large-scale pharmacoepidemiological analyses are feasible within existing NHS infrastructure. International experience further supports this approach. Nordic countries have long established national health data infrastructures that enable linkage of patient level datasets across prescribing, hospital care, microbiology and mortality records. Sweden, for example, maintains comprehensive population-based registers including the National Patient Register and the Swedish Prescribed Drug Register, which have supported extensive pharmacoepidemiological research on antimicrobial use and treatment outcomes.^31^ Denmark has similarly developed highly integrated national registries, including the Danish National Hospital Medication Register, which captures administration level prescribing data for hospitalized patients.^18^ These systems demonstrate how routinely collected patient level data can be used to evaluate medicines use and outcomes across entire populations, providing valuable real-world evidence that complements clinical trial findings. These examples reinforce the value of virtual and semi-virtual registry models and support the development of UKAR:V within the Scottish context. However, linkage-based systems also have limitations. Routine datasets often contain less clinical detail, including resistance mechanisms, than prospectively collected registry data, and several variables require operational definitions that may influence classification of treatment episodes, infection timing and outcomes. Such choices can introduce misclassification and highlight the need for sensitivity analyses to assess robustness. These constraints are inherent to studies based on routinely collected electronic data and should be considered when interpreting findings from UKAR:V.

### Interpretation of outcomes

Although readmission, relapse and mortality outcomes were successfully captured, their interpretation should remain cautious given small sample sizes and potential confounding. The variation observed across RLAs likely reflects underlying case mix and infection severity rather than differences in drug performance. Gram-negative RLAs such as ceftazidime/avibactam and cefiderocol were often initiated in intensive care, consistent with their use in critically ill patients with multidrug-resistant infections. In contrast, dalbavancin was typically used in lower-acuity or step-down care for chronic or device-related infections where recurrence and readmission remain common. These contextual differences emphasize the need to interpret outcome data in relation to underlying clinical complexity. UKAR:V demonstrates how routine data linkage can capture both outcomes and markers of disease severity, such as ICU initiation, offering a more nuanced view of real-world treatment pathways than conventional registries. While findings are descriptive and constrained by small numbers, they provide a foundation for future inferential analyses that can separate treatment effects from patient- and system-level factors. It is also important to recognize that, given the primary objective of this study was to demonstrate the feasibility and surveillance capability of the UKAR:V platform rather than to conduct diagnosis-specific effectiveness evaluations, the current analysis did not attempt to attribute hospital readmissions to specific infection diagnoses. As the UKAR:V dataset expands, future analyses will aim to link antimicrobial exposure to diagnosis-specific clinical pathways using diagnostic coding and infection-related variables, enabling more detailed evaluation of outcomes for conditions such as osteomyelitis, prosthetic joint infections and device-related infections.

### Policy and stewardship implications

UKAR:V demonstrates how national electronic health data can be used to support antimicrobial surveillance in line with the UK AMR National Action Plan and WHO GLASS priorities.^1,32^ The platform provides a mechanism for data-driven feedback to stewardship teams and for evaluating interventions such as formulary changes, guideline updates and innovative funding approaches, including the NICE subscription model.^9^ In Scotland, antimicrobial stewardship activities are coordinated nationally through the SAPG^14^ and surveillance systems such as UKAR:V can therefore provide valuable real-world evidence to inform stewardship activities led by SAPG, including monitoring uptake of newly licensed antimicrobials, evaluating adherence to treatment guidance and supporting optimization of prescribing policies. More broadly, real-world surveillance data from platforms such as UKAR:V can contribute to international antimicrobial stewardship initiatives, including the WHO Access, Watch and Reserve (AWaRe) classification framework, which aims to guide responsible antibiotic use globally.^33^ Evidence generated from linked national datasets can help inform local adaptations of such frameworks and support future updates to national treatment guidelines. With further development, UKAR:V could evolve into a core national system for continuous real-world antimicrobial surveillance, complementing and aligning with the UKAR:H to ensure integration of both detailed clinical information and large-scale population coverage. Over time, this combined approach could form a unified, data-driven intelligence framework to guide stewardship practice, inform reimbursement policy and strengthen evidence-based antimicrobial decision-making across the UK.

### Strengths and limitations

This study benefits from the use of high-quality, deterministically linked datasets that capture actual administration rather than prescribing intent and combine prescribing, microbiology and outcome data across several Scottish health boards. These features extend analytical depth beyond that achievable with prescribing datasets alone. A key limitation is that HEPMA was only implemented across six health boards during the study period, covering around 65% of the Scottish population, although national coverage has since expanded to over 91%, enhancing future representativeness. Small numbers for several RLAs limited comparative analyses, and variation in laboratory data availability may have underestimated microbiology-guided prescribing and did not include organism-level resistance mechanism information. Interpretation of hospital stay for dalbavancin also requires caution because HEPMA does not distinguish Outpatient Parenteral Antimicrobial Therapy (OPAT) from inpatient administration, meaning some recorded admissions may reflect outpatient dosing. The high proportion of apparent concomitant antibiotic use partly reflects our operational definition, whereby any overlap in administration, including same-day transitions, was counted as co-administration; future analyses will test alternative overlap windows to improve specificity. Microbiology data were available for only 35% of patients, partly due to the 7-day window applied around RLA initiation; while extending this to 7–14 days could have increased capture, it would also increase misclassification by including cultures unrelated to the index infection. We also did not restrict analyses to invasive or infection site–concordant specimens, which may have reduced clinical relevance. Although susceptibility data were available, detailed resistance mechanism information was not routinely captured, in part because susceptibility to last-line RLAs is often not tested or reported when organisms remain susceptible to first-line agents. These limitations may have contributed to incomplete or misclassified microbiology associations. Despite these constraints, the study demonstrates robust linkage, high data completeness for core variables and strong feasibility, providing a solid foundation for national expansion of the virtual registry.

### Conclusion

The UKAR:V study shows that routinely collected electronic prescribing and clinical data can support comprehensive, real-world surveillance of RLAs. The platform provides a practical mechanism to monitor uptake, use and outcomes of agents introduced through innovative reimbursement models such as the NICE subscription scheme, enabling ongoing assessment of their clinical value and stewardship impact. Although current analyses are descriptive and limited by small sample sizes, the findings demonstrate the feasibility of this approach and its potential to inform regulatory, clinical and economic decision-making. The ability of the system to capture prescribing patterns, microbiology findings, clinical outcomes and temporal prescribing trends across multiple health boards highlights the potential of linkage-based virtual registries to support continuous antimicrobial surveillance using routinely collected healthcare data. UKAR:V offers a scalable model for countries aiming to combine digital health infrastructure with antimicrobial stewardship in the context of rising resistance and evolving payment models. As HEPMA coverage increases, the platform could develop into a primary national mechanism for continuous antimicrobial surveillance, while maintaining alignment with the UKAR:H to preserve detailed clinical insights. Together, these systems could underpin a coordinated, data-driven national intelligence framework to support stewardship, guide reimbursement policy and strengthen the UK’s contribution to global AMR control efforts.

## Transparency declarations

Most authors have no conflicts of interest to declare, except for I.B. who reports investigator-initiated research funding from Shionogi B.V. and Pfizer; honoraria and travel grants from Shionogi B.V., Menarini UK and Tillotts Pharma; and participation in advisory boards for GSK, InfectoPharm and Eumedica. Additionally, R.A.S. has received honoraria for advising or speaking at educational events for NAPP/Mundipharma, Menarini, Pfizer, InfectoPharm, Advanz and Shionogi. For the purpose of open access, the author has applied a Creative Commons Attribution (CC BY) licence to any Author Accepted Manuscript.
